# An insight into the vaginal microbiome of infertile women in Bangladesh using metagenomic approach

**DOI:** 10.3389/fcimb.2024.1390088

**Published:** 2024-07-09

**Authors:** Zahid Hasan, Michael Netherland, Nur A. Hasan, Nurjahan Begum, Mahmuda Yasmin, Sangita Ahmed

**Affiliations:** ^1^ Department of Microbiology, University of Dhaka, Dhaka, Bangladesh; ^2^ EzBiome Inc., Gaithersburg, MD, United States

**Keywords:** female infertility, metagenomic analysis, CST typing, species diversity, vaginal microbiome

## Abstract

**Introduction:**

The dysbiosis of vaginal microbiota is recognized as a potential underlying factor contributing to infertility in women. This study aimed to compare the vaginal microbiomes of infertile and fertile women to investigate their relationship with infertility.

**Methods:**

Metagenomic analysis was conducted on samples from 5 infertile and 5 fertile individuals using both amplicon 16S and metagenomics shotgun sequencing methods.

**Results and discussion:**

In the infertile group, the bacterial community was primarily represented by three major bacterial genera: *Lactobacillus* (79.42%), *Gardnerella* (12.56%) and *Prevotella* (3.33%), whereas, the fertile group exhibited a more diverse composition with over 8 major bacterial genera, accompanied by significantly reduced abundance of *Lactobacillus* (48.79%) and *Gardnerella* (6.98%). At the species level, higher abundances of *L. iners, L. gasseri* and *G. vaginalis* were observed in the infertile group. Regarding the microbiome composition, only one fertile and two infertile subjects exhibited the healthiest Community State Types, CST-1, while CST-3 was observed among two infertile and one fertile subject, and CST-4 in three other fertile and one infertile subject. Overall, alpha diversity metrics indicated greater diversity and lower species richness in the control (fertile) group, while the infertile group displayed the opposite trend. However, beta-diversity analysis did not show distinct clustering of samples associated with any specific group; instead, it demonstrated CST-type specific clustering. Shotgun metagenomics further confirmed the dominance of Firmicutes, with a greater abundance of *Lactobacillus* species in the infertile group. Specifically, *L. iners* and *G. vaginalis* were identified as the most dominant and highly abundant in the infertile group. Fungi were only identified in the control group, dominated by *Penicillium citrinum* (62.5%). Metagenome-assembled genomes (MAGs) corroborated read-based taxonomic profiling, with the taxon *L. johnsonii* identified exclusively in disease samples. MAG identities shared by both groups include Shamonda orthobunyavirus, *L. crispatus*, Human endogenous retrovirus K113, *L. iners*, and *G. vaginalis*. Interestingly, the healthy microbiomes sequenced in this study contained two clusters, *Penicillium* and *Staphylococcus haemolyticus*, not found in the public dataset. In conclusion, this study suggests that lower species diversity with a higher abundance of *L. iners, L. gasseri* and *G. vaginalis*, may contribute to female infertility in our study datasets. However, larger sample sizes are necessary to further evaluate such association.

## Introduction

Infertility is a major worldwide health issue, impacting millions of individuals of reproductive age. Globally one in six couples of reproductive age suffers from subfertility effects (Farquhar et al., 2019) and experience a stressful marital life (Andrews et al., 1991). A global demographic study by the World Health Organization (WHO), identified a higher prevalence of infertility in certain developing regions of the world, including Bangladesh (WHO, n.d). Among the different reasons proposed to be responsible for infertility, bacterial vaginosis which may upset the natural microflora of the genital tract has been identified as a key factor (Ravel et al., 2021).

Vaginal infections can be caused by bacteria, fungi, parasites or viruses (Asmah et al., 2017; Lebeau et al., 2022). Of these, bacterial vaginosis (BV) is the most common vaginal infection among women of 15 to 44 years of age (Javed et al., 2019). A diverse community of anaerobic and facultative bacteria are associated with bacterial vaginosis, which may include *Gardnerella vaginalis, Atopobium vaginae, Megasphaera types, Leptotrichia amnionii, Sneathia sanguinegens, Porphyromonas asaccharolytica, Prevotella* species*, Peptostreptococcus* spp.*, Aerococcus, Anaerococcus, Gemella*, and *Veillonella* genera (Fredricks et al., 2005; Muzny et al., 2018). These pathogens may cause infertility through a variety of mechanisms, including disruption of the healthy vaginal microbiota, immune activation, inflammation, toxin production, increasing susceptibility to sexually transmitted infection and pelvic inflammatory disease (PID) (Ravel et al., 2021). In particular, reproductive tract infections caused by certain bacteria such as *Chlamydia trachomatis*, *Neisseria gonorrhoeae*, *Ureaplasma urealyticum*, *Gardnerella vaginalis* and *Trichomonas vaginalis* have well established link with clinical manifestations like pelvic inflammatory disease (PID), chronic pelvic pain, ectopic pregnancy, and infertility (Ruggeri et al., 2016; Tsevat et al., 2017; Moragianni et al., 2019). The immunologic and inflammatory changes might also disrupt endometrial function and interfere with embryo implantation and development (Park et al., 2016).

Compared to fertile women, bacterial vaginosis has been found to be three times more prevalent among infertile women, suggesting a direct link between this infection and infertility (Ravel et al., 2021). In addition to potentially increasing the likelihood of infertility, bacterial vaginosis has been related to reproductive failures, such as premature birth and late fetal loss (Ralph et al., 1999). Research also indicates that the agglutinating impact of specific microorganisms such as *E. coli* on sperm may play a noteworthy role in determining fertility outcomes (Kaur and Prabha, 2014).

Colonization of pathogenic microorganisms in the vagina is usually prevented by the vaginal commensal microbiota which consists of a very complex community of microorganisms and is typically dominated by *Lactobacillus crispatus*, *L. gasseri*, *L. jensenii*, and *L. iners* (Moragianni et al., 2019). Dysbiosis of this vaginal flora leads to colonization by pathogenic organisms, leading to complications like infertility (Findeklee et al., 2023). Therefore, to assess the risk of vaginal infection, health workers follow a community state type (CST) categorization of cervical microbiota based on the dominant commensal bacterial species. CST I, characterized by *L. crispatus* dominance, is linked to the lowest risk for bacterial vaginosis (BV), sexually transmitted infections (STIs), and infertility (Al-Nasiry et al., 2020). CST II, dominated by *L. gasseri*, has variable effects on vaginal health, with some studies suggesting a reduced incidence of BV and STIs (Ravel et al., 2011; Al-Nasiry et al., 2020). CST III, dominated by *L. iners*, is correlated with an elevated likelihood of BV and STIs (Ravel et al., 2011). Whereas, CST IV, characterized by a varied bacterial population typically led by *Gardnerella vaginalis*, is associated with an increased risk of BV, STIs, and premature birth (Ravel et al., 2011). On the other hand, CST V, dominated by *L. jensenii*, is an uncommon but beneficial state inhibiting pathogenic bacteria through lactic acid production (Amabebe and Anumba, 2018).

In Bangladesh, female infertility is a significant issue, affecting 15% of women of reproductive age, which is the highest rate among South Asian nations (Magdum et al., 2022). Additionally, between 2007 and 2014, approximately 11.1% to 13.4% of women of childbearing age in Bangladesh reported experiencing symptoms of reproductive tract infections (Feng et al., 2021). However, to date, no comprehensive research has been conducted to explore the relationship between vaginal dysbiosis with infertility in Bangladesh. Therefore, the current study aimed at investigating the vaginal microbiome of a small set of fertile and infertile women to assess any possible association between vaginal microbiome and infertility. To this end, we employed both 16S amplicon sequencing and shotgun metagenomics to study the taxonomic and functional landscapes of the vaginal microbiome.

## Materials and methods

### Study population

This study involved 10 women, divided into two groups: the diseased group (infertile) and the control group (fertile), each comprising 5 women. The diseased group sought treatment at the Department of Reproductive Endocrinology & Infertility of Bangabandhu Sheikh Mujib Medical University (BSMMU) and control samples were collected from women who had given birth within the last 6 months. Vaginal swabs were collected from each participant. All participants in the study were aged between 22 and 31 years.

The samples were collected by a specialist doctor following standard procedures, with informed consent obtained from the participating women. The participant was put in the lithotomy position, ensuring her privacy. With aseptic precaution, a sterile disposable Cuscos speculum was fixed into the vagina. Using a sterile cotton swab, secretions from the posterior fornix were collected. The swab was placed in a sterile tube and was immediately transferred to the lab for investigation. In case of any delay, the sample was kept at -20° in a lab refrigerator. Ethical clearance was also obtained from the respective authority.

### DNA extraction

The samples were transferred to the laboratory following standard guidelines. Total DNA extraction was carried out using the QIAGEN DNeasy Blood & Tissue kit, following the manufacturer’s instructions. Subsequently, the extracted DNA from all samples underwent concentration and purity checks using NanoDrop™ 2000 (Thermo Scientific, USA). The extracted DNA was then preserved at a temperature of -20°C.

### 16S rRNA amplicon sequencing

To analyze the vaginal microbial composition, the 16S V3-V4 region of the bacterial rRNA gene was sequenced at EzBiome Inc (Gaithersburg, MD, USA). Purified DNA was PCR amplified targeting the V3−V4 region of the bacterial 16S rRNA gene (341F and 805R primers) using Phusion Human Specimen Direct PCR Kit (Thermo Fisher Scientific). PCR products were then sequenced using an Illumina MiSeq sequencing system (Illumina, San Diego, USA) as previously described (Lee et al., 2020; Seong et al., 2020; Brumfield et al., 2022). Sequence data QC and taxonomic profiling were carried out using the EzBioCloud microbiome taxonomy profiling platform as described elsewhere (Yoon et al., 2017). Comprehensive profiling of the microbiome characteristics including ecological characteristics, covariate analysis, subsampling, categorization and evaluation of microbial traits, and evaluation of health-related microbial characteristics etc. were performed and reported following the bioinformatics framework described elsewhere (Yoon et al., 2017). Briefly, microbial richness was measured by ACE, Chao1, Jakknife and the number of OTUs found in the microbiome taxonomic profile (MTP) index. The Shannon, Simpson and Phylogenetic α-diversity metrics were applied to estimate the diversity for each group using the Wilcoxon rank-sum test. Beta diversity was calculated with Jansen-Shannon, Bray-Curtis, UniFrac and Generalized UniFrac distances based on the taxonomic abundance profiles. Permutational multivariate analysis of variance (PERMANOVA) was applied to measure the statistical significance of β-diversity. Different groups were clustered with Principal Coordinates Analysis (PCoA) based on the abundance Jaccard distance metric. Kruskal-Wallis H test, LEfSe and Taxon XOR analysis were performed to determine enrichment in the assigned taxonomic and functional profiles between groups. Statistically significant differences were determined by P values less than 0.05.

### Shotgun metagenomic sequencing

To conduct an in-depth analysis as well as to assess the non-bacterial microbial community of the vaginal ecosystem, whole-metagenome shotgun sequencing was employed. All ten samples were sequenced at EzBiome Inc (Gaithersburg, MD, USA). The concentration of genomic DNA was measured using the Qubit Fluorometer dsDNA DNA quantification System (ThermoFisher, USA) followed by the use of 50ng-1μg of genomic DNA for library construction using NEBNext® Ultra™ II FS DNA Library Prep Kit for Illumina®. The libraries were quantified and qualified using the D1000 ScreenTape on an Agilent 2200 TapeStation instrument. The libraries were normalized and pooled for multiplexed sequencing on an Illumina HiseqX10 sequencer (Illumina, San Diego, CA, USA) using the pair-end 150bp run format. All ten samples resulted in an average sequencing depth of ~ 40 million reads per sample. Furthermore, we have included 10 publicly available vaginal metagenomic samples of Fijian women published elsewhere (Bommana et al., 2022). Their average sequencing depth was 3,484,898 reads with an average sequence length of 203bp.

### Shotgun taxonomic and functional profiling

The profiling process started by surveying the potential presence of bacterial and archaeal species for each raw metagenomic sample read by using Kraken2 (Wood et al., 2019) and a pre-built core gene database (Chalita et al., 2020) containing k-mers (k=35) of reference genomes obtained from the EzBioCloud database (Yoon et al., 2017). Fungi and Viral full genomes from NCBI’s RefSeq (https://www.ncbi.nlm.nih.gov/refseq/) were also added to the Kraken2 database. After acquiring a list of candidate species, a custom bowtie2 (Langmead and Salzberg, 2012) database was built utilizing the core genes and genomes from the species found during the first step. The raw sample was then mapped against the bowtie2 database using the –very-sensitive option and a quality threshold of phred33. Samtools (Li et al., 2009) was used to convert and sort the output bam file. Coverage of the mapped reads against the bam file was obtained using Bedtools (Quinlan and Hall, 2010). Then, to avoid false positives, using an in-house script, we quantified all the reads that mapped to a given species only if the total coverage of their core genes (archaea, bacteria) or genome (fungi, virus) was at least 25%. Finally, species abundance was calculated using the total number of reads counted and normalized species abundance was calculated by using the total length of all their reference. For each sample, functional annotations were obtained by matching each read, using DIAMOND (Buchfink et al., 2015), against the KEGG database (Kanehisa et al., 2017). DIAMOND was executed using the blastx parameter, which converts each metagenomic read into multiple amino acid sequences by generating all six open reading frame variations, and then matches it against the pre-built KEGG database. If a read had multiple KEGG hits, the top hit was always used. After quantifying all the KEGG orthologs present, minpath (Ye and Doak, 2009) was used to predict the presence of KEGG functional pathways. To increase the analytical robustness of our investigation, after initial taxonomic profiling, read counts were normalized with DESeq2’s median of ratios method (Love et al., 2014). Additionally, to better describe the species diversity within each cohort (diseased, control, public) of datasets, the average relative abundance within each kingdom is reported separately.

### Taxonomy independent analysis

Metagenomic assembly of diseased and control samples was employed as an additional perspective to investigate the difference between the cohorts. Each sample was assembled individually with SPAdes v3.15.5, using the –meta flag, followed by clustering of contigs with VizBin, and manual extraction of clusters. Each cluster, constituting a metagenomically-assembled genome (MAG), was checked for quality with CheckM. MAGs not identified at the kingdom level or as “root” by CheckM were identified at the species level with TrueBacID (Ha et al., 2019). All other MAGs were used for viral profiling. Viral investigation was performed by aligning reads to corresponding MAGs with Bowtie2 and taxonomic profiling was performed on captured reads as described above. MAGs were excluded if there were more than 3 taxa profiled, and any remaining MAGs were labeled for the taxa or taxon with the highest relative abundance. The contigs for the MAGs identified by TrueBacID and those profiled by metagenomic methods were combined, analyzed with VizBin, and the coordinates were used to plot them using plotly.

### Confounding variables analysis

To correlate metadata with changes in microbiome unconstrained ordination was performed on the Bray-Curtis dissimilarity matrix of normalized species counts with the cmdscale function in R. The metadata variables were fit to the ordination using the ‘envfit’ function in the vegan package. The ‘strata’ option was used to constrain analysis permutations by cohort status (control/diseased). FDR correction of p-values was applied for multiple hypothesis testing.

## Results

### Microbiome profiling using 16S amplicon sequencing

**Figure 1:**
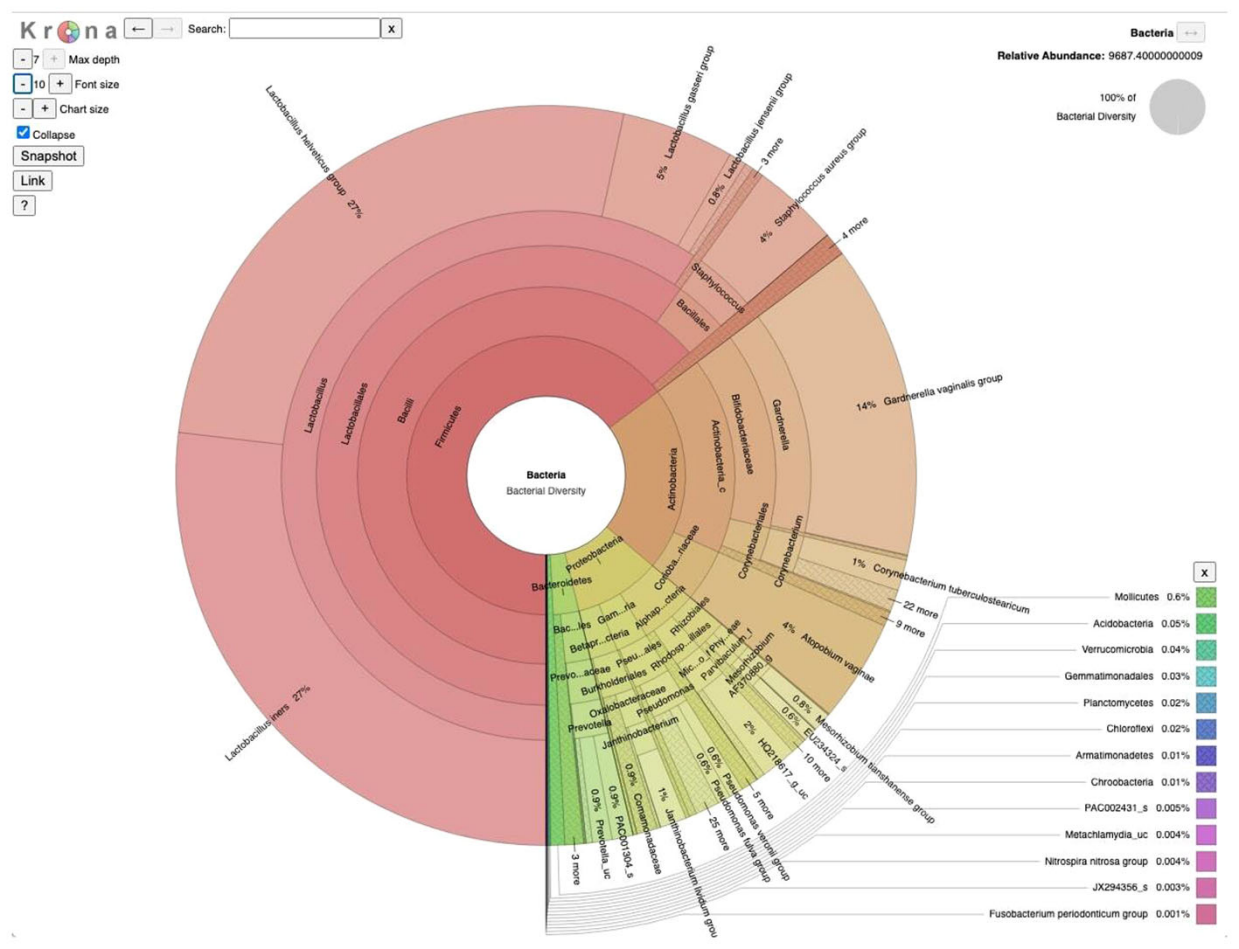
Taxonomic composition of vaginal microbiome in Bangladesh: A visualization of the total microbial community from 10 vaginal samples (5 infertile and 5 fertile) collected at a tertiary hospital in Bangladesh, presented using a Krona multilayered pie chart established at the species level derived from 16S amplicon sequencing data.

16S amplicon sequencing was used to study the microbial community associated with the vaginal samples collected from 10 study subjects. Bioinformatic analysis of the 16S datasets using the EzBioCloud platform (Yoon et al., 2017) revealed that 365 bacterial genera representing 159 families and 20 bacterial phyla constitute the vaginal microbiome of the study subjects with four major phyla comprising over 99% of community composition (**Figure 1**). Overall, Firmicutes appeared to be the dominant phylum (~64.4%) of the community followed by Actinobacteria (21.7%), Proteobacteria (10.1%), and Bacteroidetes (2.9%), whereas Actinobacteria (infertile subject ZH5 and fertile subject C1) and Proteobacteria (fertile subject C3), instead of Firmicutes, appeared as the dominant phylum in three subjects. Six genera made up > 85% of average community composition of the vaginal microbiome, with *Lactobacillus* (57.6%) being the dominant genus followed by *Gardnerella* (13.3%), *Staphylococcus* (5.2%), *Atopobium* (4.3%), *Corynebacterium* (3.0%), and *Pseudomonas* (2.2%) (**Figure 1**), whereases non-lactobacillus dominance was observed in three subjects: infertile subject ZH5 and fertile subjects C1 and C4 which constitutes *Gardnerella* (66.47%), *Atopobium* (43.33%), and *Staphylococcus* (51.65%) as the dominant member of the community.

**Figure 2:**
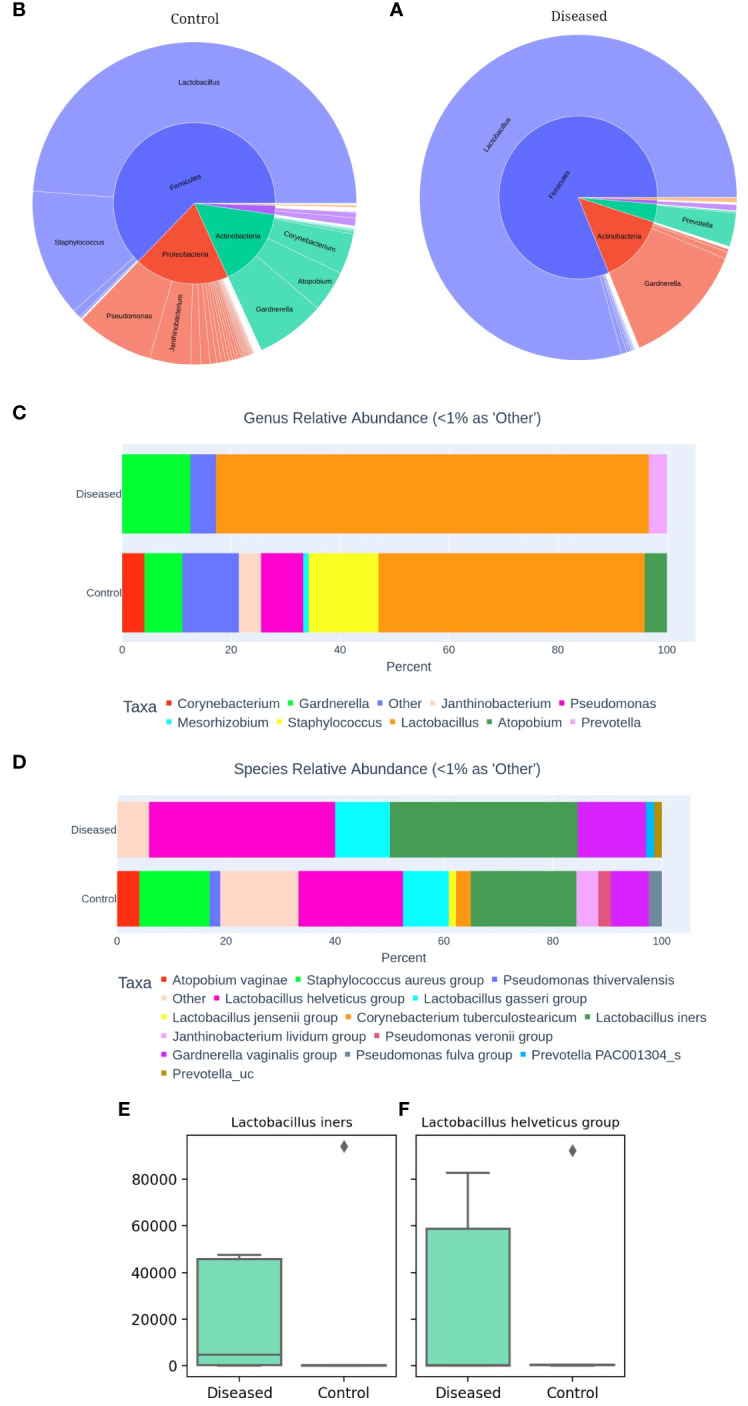
Comparative Analysis of Vaginal Microbiomes **(A)** Microbial community of Diseased (infertile) and **(B)** Control (fertile) vaginal samples. Relative abundance of dominant bacterial genera **(C)** and species **(D)**. Abundance of *Lactobacillus iners* **(E)** and *Lactobacillus helveticus* **(F)** between the two groups, as determined by 16S amplicon sequencing of 10 vaginal samples.

Next, we compared the microbial community associated with the two groups of subjects, i.e., diseased and control to investigate potential taxonomic and compositional differences, if any, between them. Comparative analysis of the two groups demonstrated somewhat distinct compositional profiles (**Figures 2A, B**) associated with diseased and control cohorts which are characterized mostly by differential abundances of four major bacterial phyla: Firmicutes, Bacteroidetes, Proteobacteria, and Actinobacteria. Firmicutes abundance was relatively higher in the diseased group (**Figure 2A**) whereas the control group demonstrated relatively higher abundances of Proteobacteria (**Figure 2B**). The bacterial community associated with the disease (infertile) group is primarily represented by three major (>1% average relative abundance, RA) bacterial genera, i.e., *Lactobacillus* (79.42%), *Gardnerella* (12.56%) and *Prevotella* (3.33%) whereases control (fertile) group comprised over 8 major bacterial genera with reduced abundance of *Lactobacillus* (48.79%) (**Figure 2C**). Furthermore, a greater abundance of *Gardnerella*, constituting 12.56% of the community composition, was observed in the diseased group, compared to that of 6.98% in the control group. Such a higher abundance of *Gardnerella vaginalis* in the diseased group may contribute to the elevated pH in the vaginal microenvironment resulting in dysbiosis or unstable environments that are prone to recurrent infections and increased risk of adverse health outcomes.

Upon closer inspection at the species level (**Figure 2D**), it becomes evident that the diseased group comprised of much fewer (n=6) bacterial species with >1% RA than that of the control group (n=12). Disease group constitutes relatively higher abundances of *L. iners* and members of *L. helveticus*, *L. gasseri* and *Gardnerella vaginalis* group compared to the control group. Interestingly, *Lactobacillus iners* and *Lactobacillus helveticus* are present as dominant species in both groups (**Figures 2E, F**). However, their abundance is higher in the diseased group, with *Lactobacillus iners* constituting 34.52% and *Lactobacillus helveticus* 34.21%, compared to 19.40% and 19.15% in the control group, respectively. While *Lactobacillus helviticus* is associated with a healthy vaginal flora, *Lactobacillus iners* is often linked to vaginal dysbiosis. Additionally, *Lactobacillus gasseri* is present in higher abundance in the disease group, constituting 10.01%, compared to the control group, which contains 8.43%. After comparing the differential abundances of *Lactobacillus* species, a bacterium that plays a critical role in the vaginal and cervical microenvironment, we next moved to characterize the associated Community State Types (CSTs) of the studied subjects (Ravel et al., 2011). CST typing of the microbiome profiles shows (**Table 1**) that only one control subject (C2) represents the healthiest type, CST-1, which is known to be associated with optimal vaginal health, whereas surprisingly two out of the five diseased (infertile) subjects (i.e., ZH3 and ZH4) had the healthiest CST-1 type. Type 3 which is characterized by the dominance of *Lactobacillus iners* is observed among two diseased (ZH1 & 2) and one control (C5) subject. Type 4 is typically characterized by low abundance of Lactobacilli and high diversity and dominance of other bacteria. CST-4 can be associated with dysbiosis and is indicative of a less stable environment prone to infections. Interestingly, the microbiome profile of three healthy subjects (C1, C3 & C4) appears to be of Type 4, whereas only one diseased subject (ZH5) had CST-4 (**Table 1**).

**Table 1:** Community State Type (CST) profiles of the microbiome.

Subjects	Dominant Species (includes species with >15% relative abundance)	Relative Abundance	CST Type
ZH1	*Lactobacillus iners*	90.90%	CST-3
ZH2	*Lactobacillus iners*	68.78%	CST-3
ZH3	Lactobacillus helveticus group*	96.15%	CST-1
ZH4	*Lactobacillus helveticus group*	64.86%	CST-1
ZH5	*Gardnerella vaginalis group; Lactobacillus gasseri group*	66.43%; 29.08%	CST-4A
C1	*Atopobium vaginae;* *Gardnerella vaginalis group;* *Lactobacillus gasseri group*	43.33%; 36.46%; 18.21%	CST-4B
C2	*Lactobacillus helveticus group*	93.87%	CST-1
C3	*Multiple* Non*-Lactobacillus species (mostly Proteobacteria)*	5.21%-15.24%	CST-4C
C4	*Staphylococcus aureus group;* *Corynebacterium tuberculostearicum*	51.60%; 16.31%	CST-4C
C5	*Lactobacillus iners*	95.94%	CST-3

*Lactobacillus helveticus group includes *L. crispatus.*

* *Lactobacillus helveticus* group includes *L. crispatus*. Further investigation at the sequence level indicated a substantial number of reads were associated with *L. crispatus*, which has been confirmed by Shotgun analysis as well.

### Alpha diversity analysis

**Figure 3:**
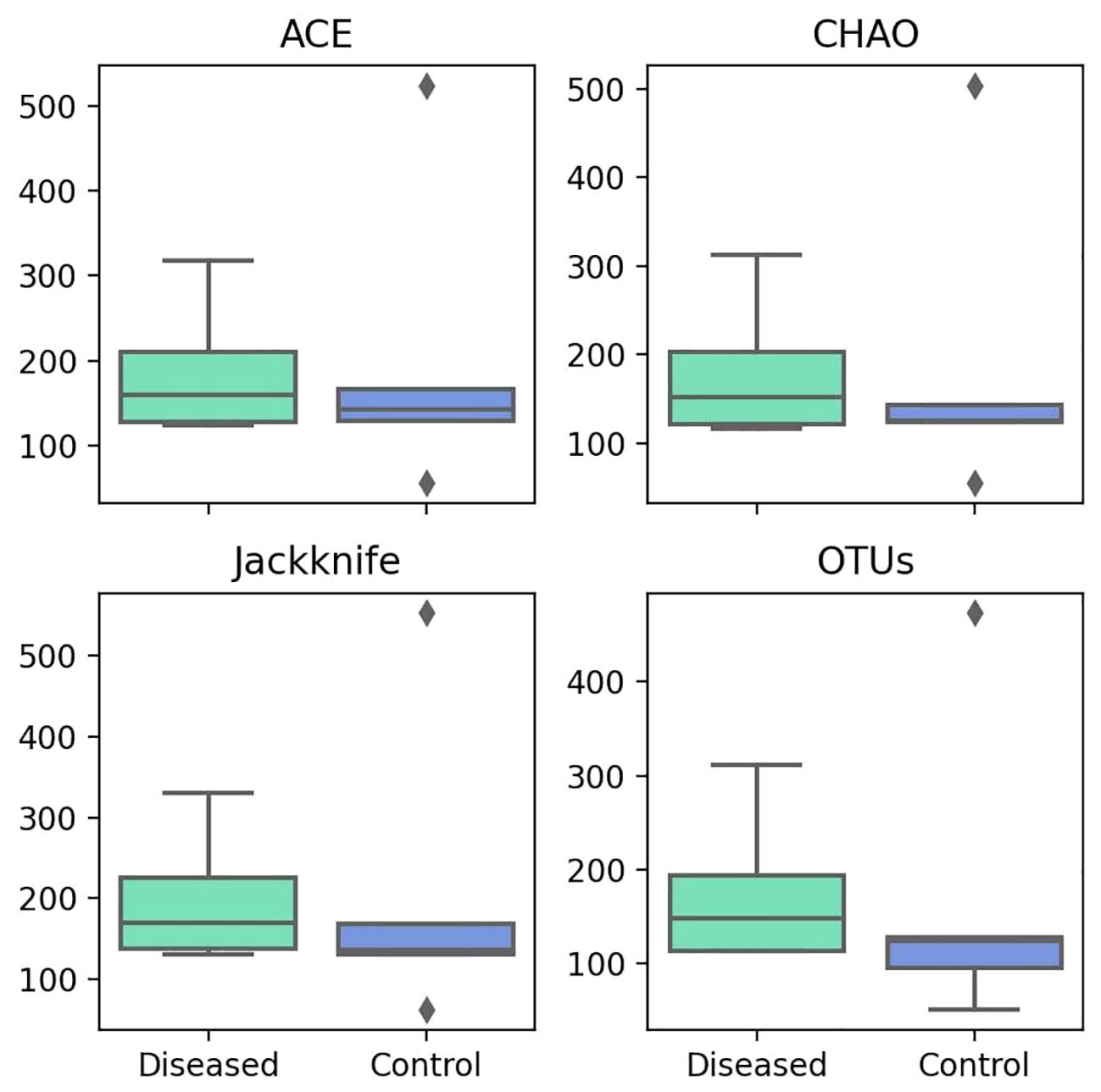
Comparison of Different Alpha Diversity Indices (Species Richness) between Diseased (Infertile) and Control (Fertile) Groups using the Wilcoxon rank-sum test: ACE index (P=0.917), Chao1 index (P=0.754), Jackknife index (P=0.347), and The number of OTUs found in MTP (P=0.465).

The alpha diversity metrics, including ACE, Chao1, Jackknife, and the number of OTUs, serve as indicators of species richness within the samples. The higher the value, the greater the richness. The median values for Ace, Chao1, Jackknife, and the number of OTUs in the diseased group are 159.40, 151.39, 169, and 148, respectively (**Figure 3**). Conversely, in the control group, the median values are 142.91, 125.32, 136, and 124, respectively. We consistently measured greater species richness in the disease group compared to the control group.

**Figure 4:**
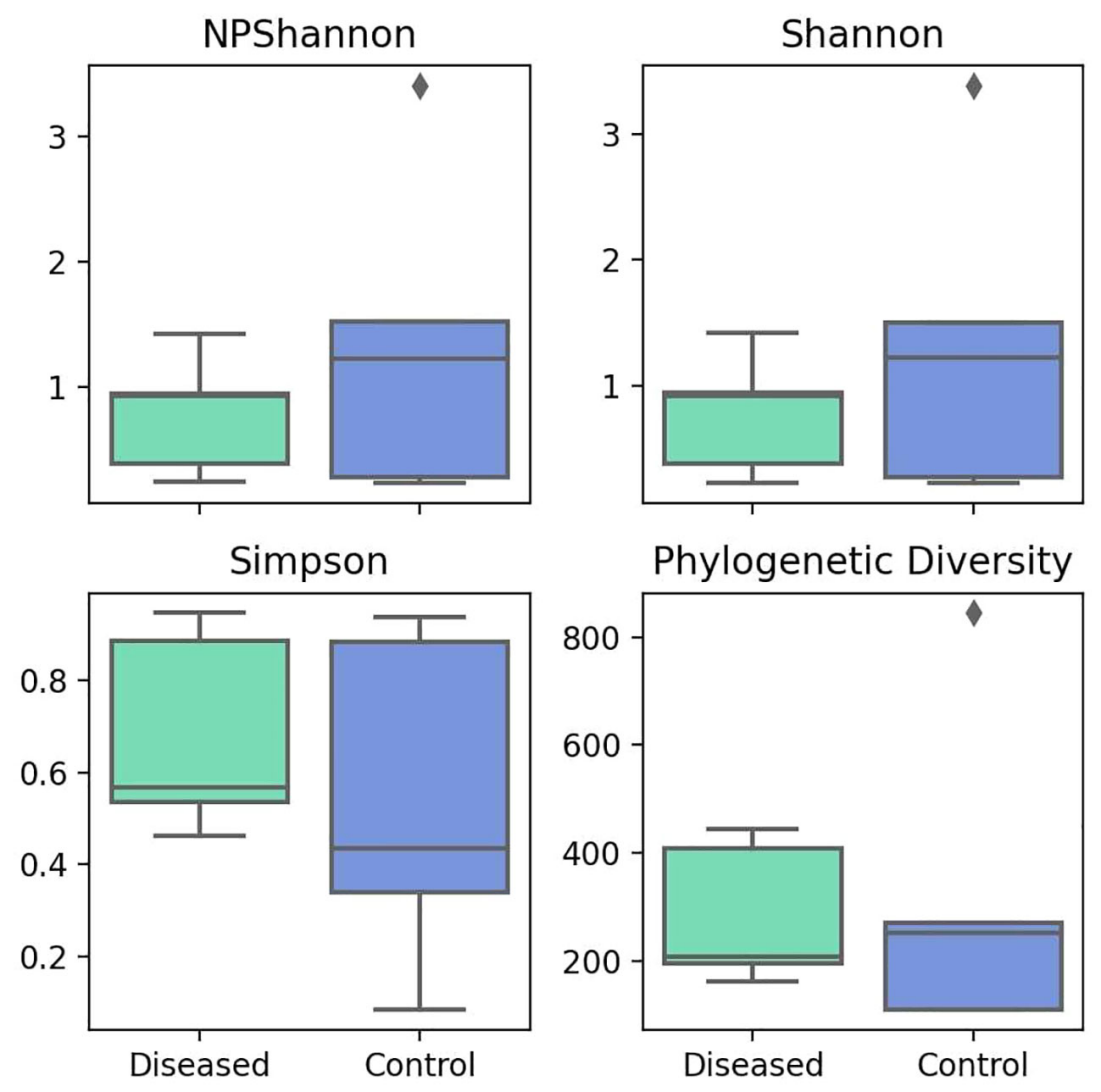
Comparison of Different Alpha Diversity Indices (Diversity Index) between Diseased (Infertile) and Control (Fertile) Groups using the Wilcoxon rank-sum test: NPShannon index (P=0.602), Shannon index (P=0.602), Simpson index (P=0.251), and Phylogenetic Diversity (P=0.754).

The alpha diversity indices like NPShannon and Shannon measure diversity, considering both species abundance and evenness. Higher values indicate greater diversity. The median values for both NPShannon (**Figure 4**) and Shannon (**Figure 4**) in the control group are 1.22. In contrast, the median values for the diseased group are 0.92 for NPShannon and 0.91 for Shannon. Both metrics suggest higher species diversity in the control group in terms of both species’ abundance and evenness. In the case of the Simpson value, a lower value indicates greater diversity. The disease group has a Simpson median of 0.57, while the control group has a median of 0.44, indicating greater diversity in the control group (**Figure 4**).

Taking into account the evolutionary relationships between species, the phylogenetic diversity metric indicates diversity in evolutionary lineages, where a higher value indicates greater diversity. The diseased group has a median value of 207, while the control group has a median value of 251 (**Figure 4**), indicating greater diversity in the control group.

Overall, all the alpha diversity metrics indicate greater diversity and lower species richness in the control group, whereas the disease group demonstrates lower diversity measures but greater species richness. However, no statistical significance is observed in any of the cases.

### Beta diversity analysis

**Figure 5:**
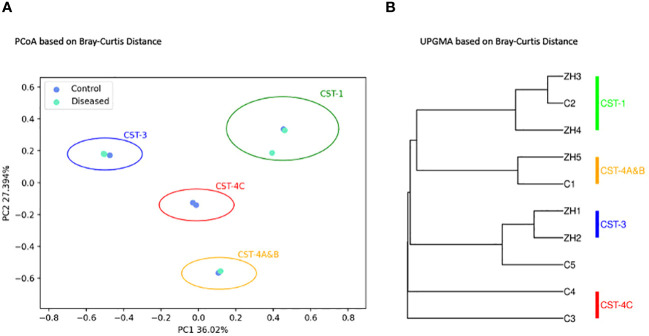
Microbial Beta Diversity Analysis between Diseased (Infertile) and Control (Fertile) Groups using the Bray-Curtis Index. **(A)** Principal Coordinates Analysis (PCoA) showing Diseased (green) and Control (blue) samples. **(B)** Unweighted Pair Group Method with Arithmetic Mean (UPGMA) clustering. The analyses are based on 16S amplicon sequencing data.

Beta diversity, which measures the variation in species composition among different samples, was analyzed using Principal Coordinates Analysis (PCoA) (**Figure 5A**) and the Unweighted Pair Group Method with Arithmetic Mean (UPGMA) (**Figure 5B**). The distances between the samples correlate with their diversity dissimilarities, where a greater distance indicates a greater dissimilarity.

As depicted in **Figures 5A**, **B**, beta-diversity analysis did not show distinct clustering of samples associated with diseased and control groups as expected. Both PCoA (**Figure 5A**) and UPGMA (**Figure 5B**) clustering demonstrates four distinct clusters of subjects characterized by the microbial composition, dominant bacterial species associated with each subject, and CST types. As shown, (**Figures 5A, B**) the clusters are well separated as they are distinguished by their community state types. The disease samples ZH1 and ZH2 are clustered together with the control sample C5, while ZH3 and ZH4 samples from the disease group are closely related to the control sample C2. Control sample C1 is clustered with the disease sample ZH5, and the two control samples, C3 and C4, are clustered separately to form another cluster representing CST-4C.

### Microbiome profiling using shotgun metagenomics

To investigate the association of the non-bacterial microbiome of the vaginal ecosystem as well as to cross validate our observations from amplicon-based bacterial community profiling, we further employed shotgun metagenomics to these set of samples and included a set of 10 publicly available metagenomic samples of Fijian women published elsewhere (Bommana et al., 2022).

**Figure 6:**
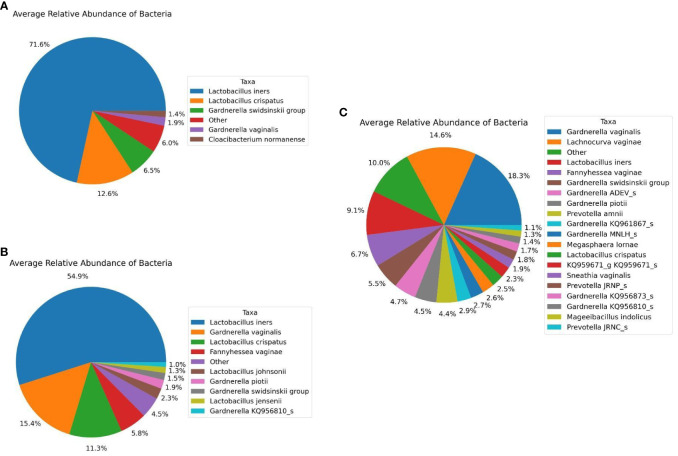
Average Relative Taxonomic Abundance of Bacteria in Each Cohort Analyzed Using Shotgun Metagenomics Analysis. **(A)** Diseased Group. **(B)** Control Group, and **(C)** Public Datasets from NCBI.

Regarding bacterial taxa constituting the diseased and control subject in our study, we observed similar trend as we observed with 16S microbiome profiling, i.e., microbiome of disease group comprises fewer bacterial species with high abundances of *Lactobacillus* species compared to that of the control group. Five major bacterial species (with > 1.0% average relative abundance) namely, *Lactobacillus iners* (71.6%), *Lactobacillus crispatus* (12.6%), *Gardnerella swidsniskii* group (6.5%), *Gardnerella vaginalis* (1.9%), and *Cloacibacterium normanense* (1.4%) (**Figure 6A**) comprises the microbiome of infertile women (diseased group). The microbiome of the control group, on the other hand, comprises *Lactobacillus iners* (54.9%), *Gardnerella vaginalis* (15.4%), *Lactobacillus crispatus* (11.3%), *Fannyhessea vaginae* (5.8%), *Lactobacillus johnsonii* (2.3%), *Gardnerella piotii* (1.9%), *Gardnerella swidsniskii* group (1.5%), *Lactobacillus jensenii* (1.3%), and *Gardnerella KQ956810_s* (1.0%) (**Figure 6B**). Interestingly, the microbiome of the public dataset (Fijian women) used in this study, demonstrated noticeable differences, i.e., greater species diversity, and relatively lower abundances of *Lactobacillus* species, compared to that of our study cohort. The microbiome of the Fijian women cohort comprises of *Gardnerella vaginalis* (18.3%), *Lachnocurva vaginae* (14.6%), *Lactobacillus iners* (9.1%), *Fannyhessea vaginae* (6.7%), *Gardnerella swidsniskii* group (5.5%), *Gardnerella ADEV_s* (4.7%), *Gardnerella piotii* (4.5%), *Prevotella amnii* (4.4%), *Gardnerella KQ961867_s* (2.9%), *Gardnerella MNLH_s* (2.7%), *Megasphaera lornae* (2.6%), *Lactobacillus crispatus* (2.5%), KQ959671_g KQ95671_s (2.3%), *Sneathia vaginalis* (1.9%), *Prevotella JRNP_s* (1.8%), *Gardnerella KQ956873_s* (1.7%), *Gardnerella KQ956810_s* (1.4%), *Mageeibacillus indolicus* (1.3%), and *Prevotella JRNC_s* (1.1%) (**Figure 6C**). Comparison of bacterial taxonomic profiles between the diseased and control groups revealed a dominance of Firmicutes, in particular, the *Lactobacillus* species in both groups but there is a greater abundance of *Lactobacillus* in diseased samples than in the controls. Similar to the 16S metagenomics analysis, *Lactobacillus iners* and *Gardnerella vaginalis* are the most dominant at the species level (**Figures 6A, B**).

**Figure 7:**
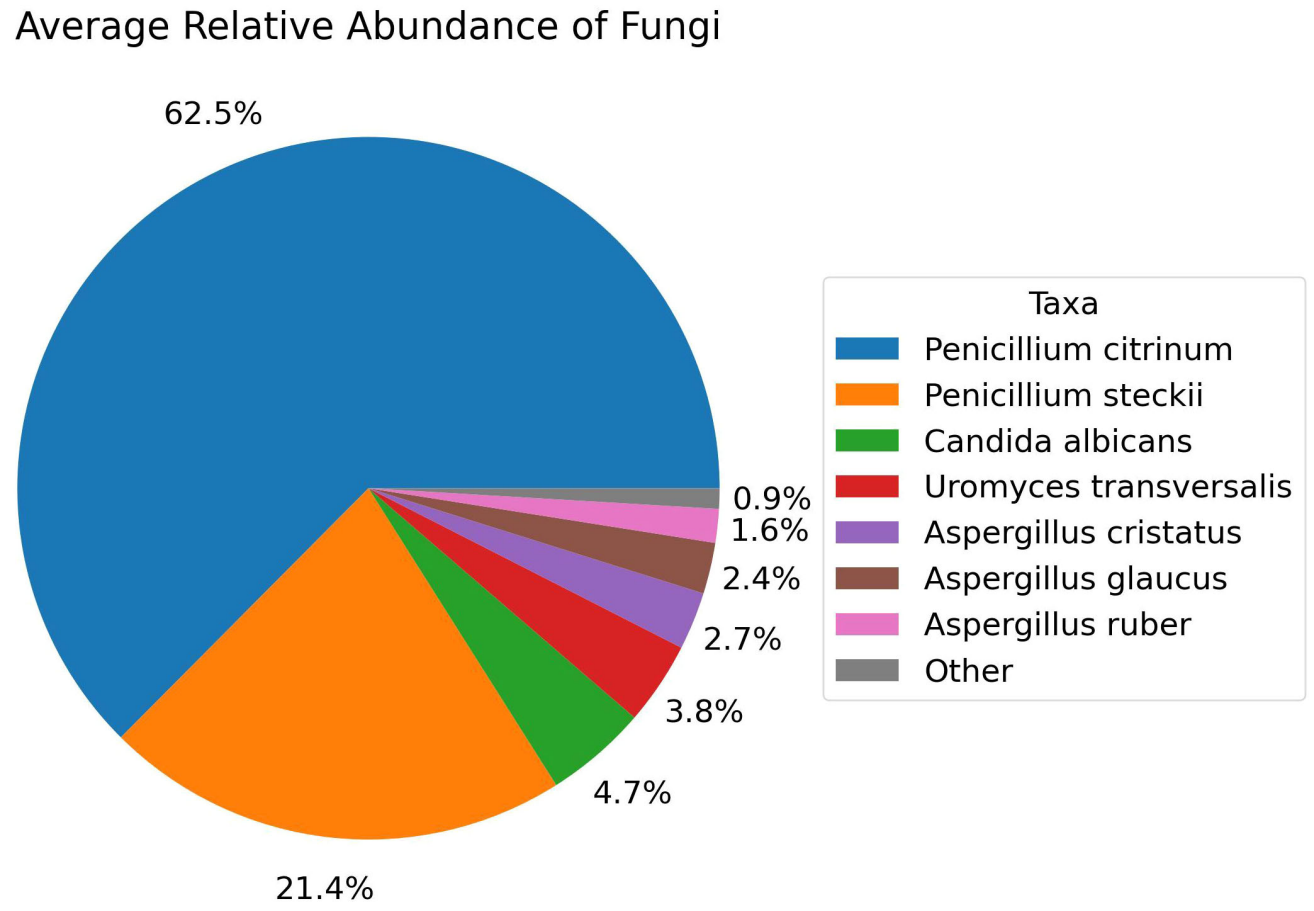
Relative Abundance of Fungal species in the Control group, Analyzed with Shotgun Metagenomics.

**Figure 8:**
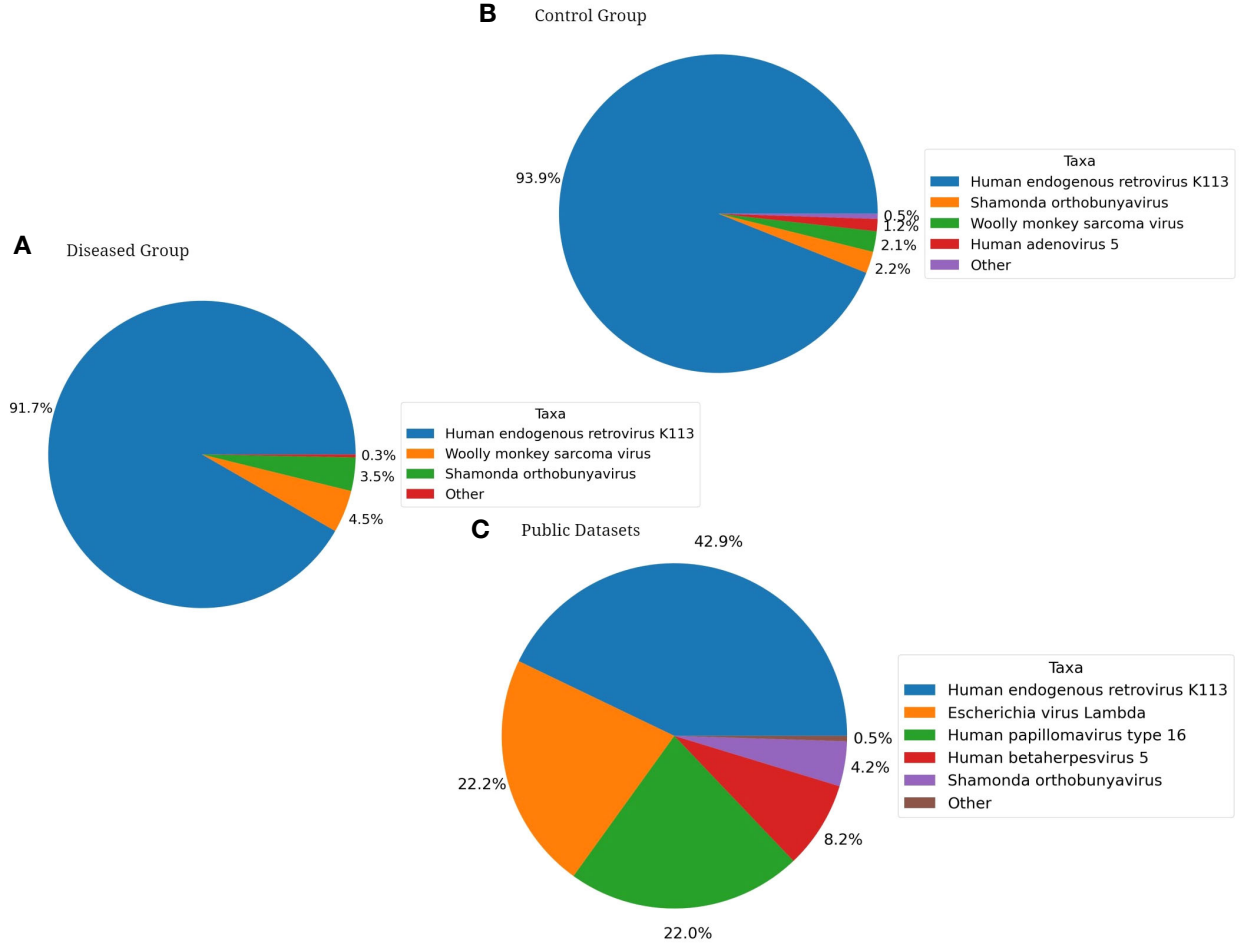
Pie Charts depicting Shotgun Metagenomics Analysis of Average Relative Taxonomic Abundance of Viruses in Each Cohort: **(A)** Diseased Group, **(B)** Control Group, **(C)** Public Dataset from NCBI.

Furthermore, when exploring for non-bacterial microbiome, Fungi were only identified in the healthy group of fertile women and among the fungal species with >1.0% average relative abundance comprises *Penicillium citrinum* (62.5%), *Penicillium steckii* (21.4%), *Candida albicans* (4.7%), *Uromyces transversalis* (3.8%), *Aspergillus cristatus* (2.7%), *Aspergillus glaucus* (2.4%), and *Aspergillus ruber* (1.6%) as part of the fertile women mycobiome (**Figure 7**). On the other hand, Viruses were identified in all three groups. Major viral taxa in the diseased group with greater than 1.0% average relative abundance consists of Human endogenous retrovirus K113 (91.7%), Wooly monkey sarcoma virus (4.5%), and Shamonda orthobunyavirus (3.5%) (**Figure 8A**). The control group consists of Human endogenous retrovirus K113 (93.9%), Shamonda orthobunyavirus (2.2%), Wooly monkey sarcoma virus (2.1%), and Human adenovirus 5 (1.2%) (**Figure 8B**). The public dataset consists of Human endogenous retrovirus K113 (42.9%), Shamonda orthobunyavirus (4.2%) (**Figure 8C**).

### Taxonomy independent analysis

**Figure 9:**
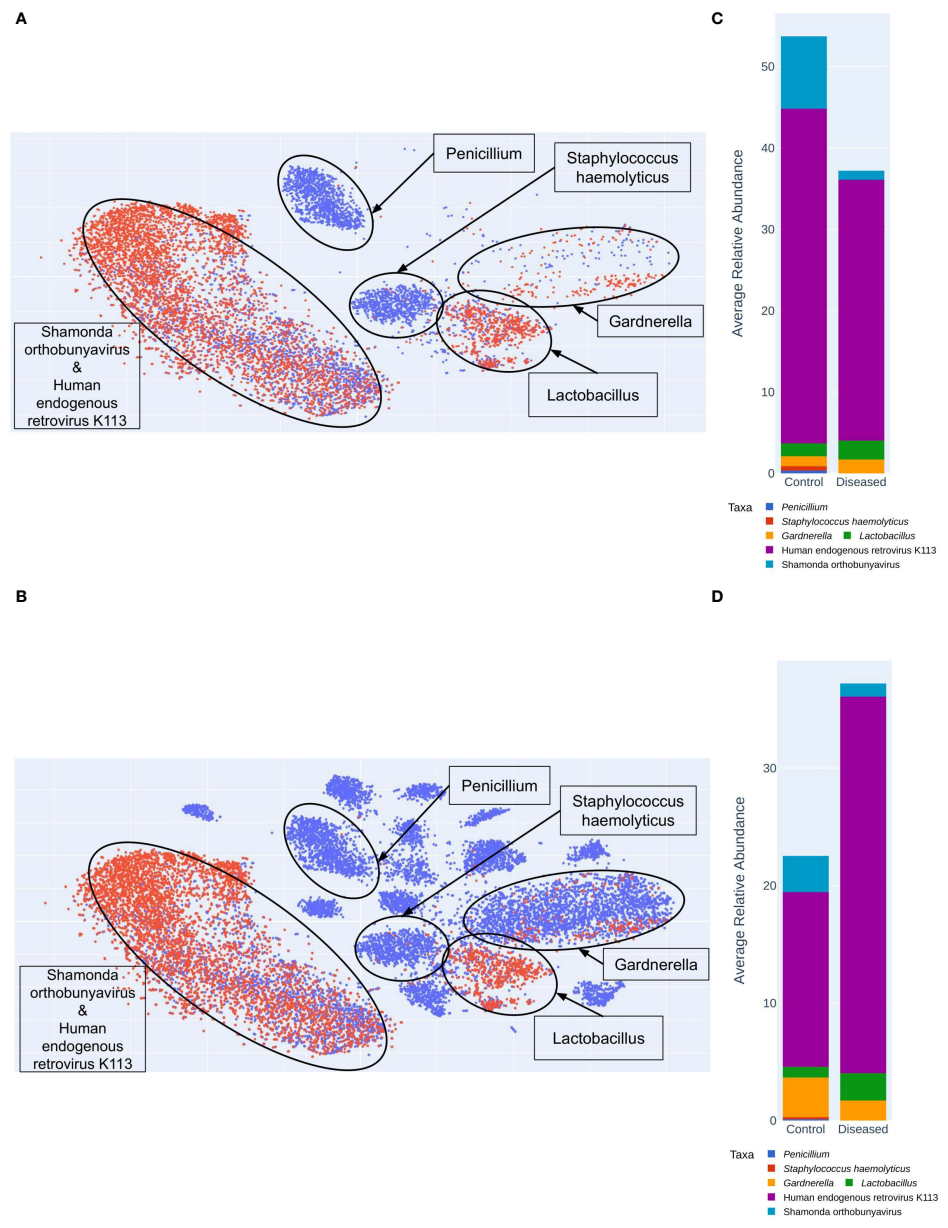
Shotgun MAG (Metagenomically Assembled Genomes) Clustering Analyzed Using VizBin. **(A)** Clustering of only Diseased (Red) and Control (Blue) samples. **(B)** MAG clustering with the additional ten control samples (Blue) from the public (NCBI) database. Average relative abundance of taxa in Control and Diseased groups **(C)** excluding the public dataset **(D)** including 10 public datasets from NCBI.

The public data helps to show the greater diversity in control vs. diseased vaginal microbiomes. Using VizBin, 72 MAGs were manually extracted based on their spatial separateness. Twenty-four MAGs were used for identification using TrueBac ID. The remaining 48 MAGs were used for read alignment and subsequent metagenomic profiling to understand their taxonomic contents. Of the read sets that were captured after alignment, only 22 yielded any results. Of these, 15 had results where 100% of the reads belonged to a single taxon, 3 were excluded due to too many taxonomic assignments, and the remaining four were labeled for the taxa or taxon with the highest relative abundance (65.7% - 99.5%). Of these 19 MAGs, 17 were identified as viral taxa, 1 was identified as *Lactobacillus iners*, and 1 was identified as genus *Penicillium*. After curation, the contigs of 43 MAGs were used for VizBin analysis and contig coordinate plotting (**Figure 9**).

We were able to extract more MAGs from the public data than the samples sequenced in this study (**Figure 9**). MAG identities for the public data include *Bifidobacterium faecale*, Human papillomavirus type 16, *Gardnerella KQ956810_s*, *Gemella massiliensis*, *Gardnerella ADEV_s*, *Porphyromonas KQ959247_s*, *Prevotella JRNC_s*, *KQ959671_g KQ959671_s*, *Mobiluncus curtisii, Porphyromonas ACLR_s*, *Fannyhessea vaginae*, *Megasphaera AFUG_s*, *Acetitomaculum ruminis*, *Sneathia vaginalis*, *Prevotella amnii*, *Metaprevotella massiliensis*, *Gardnerella swidsinskii*. MAG identities for samples in this study include: Shamonda orthobunyavirus, *Penicillium*, *Lactobacillus crispatus*, *Staphylococcus haemolyticus*, Human endogenous retrovirus K113, *Lactobacillus iners*, *Lactobacillus johnsonii*. The public dataset lacks any *Lactobacillus* MAGs, and both datasets include a MAG identified as *Gardnerella vaginalis*. MAG identities exclusive to control samples include: *Penicillium*, *Staphylococcus haemolyticus*, *Bifidobacterium faecale*, Human papillomavirus type 16, *Gardnerella KQ956810_s*, *Gemella massiliensis*, *Gardnerella ADEV_s*, *Porphyromonas KQ959247_s*, *Prevotella JRNC_s*, *KQ959671_g KQ959671_s*, *Mobiluncus curtisii*, *Porphyromonas ACLR_s*, *Fannyhessea vaginae*, Megasphaera AFUG_s, *Acetitomaculum ruminis*, *Sneathia vaginalis*, *Prevotella amnii*, *Metaprevotella massiliensis*, *Gardnerella swidsinskii*. The taxon *Lactobacillus johnsonii* is the only MAG exclusive to disease samples. MAG identities shared by control and diseased samples include Shamonda orthobunyavirus, *Lactobacillus crispatus*, Human endogenous retrovirus K113, *Lactobacillus iners*, *Gardnerella vaginalis*. Interestingly, the control microbiomes sequenced in this study contain two clusters not found in the public dataset: *Penicillium* and *Staphylococcus haemolyticus* (**Figure 9**).

### Confounding variables analysis

To test the significance of participant metadata on changes in the vaginal microbiome, we employed the function envfit in the vegan R library. We have used the variables of age, gravidity and parity to test the significance of their correlation with the ordination derived from a Bray-Curtis distance matrix of normalized species counts. The envfit analysis was performed using stratification to constrain analysis permutations by cohort status (control/diseased), as a way to control for that variable during significance testing. Results indicate that the fitted vectors of the continuous variables ‘Age’, ‘Gravidity’, and ‘Parity, do not significantly correlate with changes in the cervical microbiome in these samples.

### Discussion

Infertility research has traditionally been focused on non-infectious paradigms, often overlooking the significant role of vaginal infections and associated microbial diversity (Al-Nasiry et al., 2020). The understanding of the latter scope still remains unclear due to the complex host-microbial interaction as well as the inherent diversity and composition of vaginal microflora in different women. Moreover, factors such as race, ethnicity and locality pose additional challenges to infertility research. It is quite possible that infertility is not caused by a single population but rather by an intricate interplay of a diverse microbial population involving both pathogenic and commensal microorganisms (Campisciano et al., 2020). To elucidate this possibility, our study comparatively assessed the diverse composition of the vaginal niche in fertile and infertile females, employing a metagenomic approach to identify mutually exclusive microorganisms, whether pathogenic or not, in the subject and control sets, which could be associated with female infertility.

Our primary focus was on taxonomic characterization to correlate the vaginal microbial population with infertility. Amplicon metagenomic analysis at the genus level revealed the predominance of *Lactobacillus* in both the disease and control groups. This finding was expected, given that *Lactobacillus* species are the natural inhabitants of the vagina, crucial for maintaining a lower vaginal pH by producing lactic acid, which in turn inhibits the colonization of pathogenic microorganisms (Frąszczak et al., 2023). Interestingly, in one diseased sample, *Gardnerella* dominated, which has been previously associated with infertility (Gholizadeh et al., 2023). Conversely, two samples from the control group were dominated by *Atopobium* and *Staphylococcus*, respectively.

Given the functional diversity among various species within the same genus, it is important to note that not all *Lactobacillus* species exhibit equal potency in protective roles. Some species may be less effective in preventing dysbiosis of the vaginal flora, potentially contributing to infertility (Zheng et al., 2021). Therefore, we next focused on the species-level identification of these microorganisms. At the species level, although no clear distinction was observed between the two study groups, some important insights were retrieved. Our analysis revealed that two samples from the diseased group were dominated by *Lactobacillus iners*, two by *Lactobacillus helveticus*, and one by *Gardnerella vaginalis*, representing community state type (CST) 3, 1, and 4A, respectively. While *Lactobacillus helveticus*, indicative of CST type 1, typically signifies a healthy vaginal community (Taverniti and Guglielmetti, 2012; Pino et al., 2019), the presence of *Lactobacillus iners* (CST-3) and *Gardnerella vaginalis* (CST-4A) is associated with poor vaginal health, potentially linked to infertility (Campisciano et al., 2020; Gholizadeh et al., 2023). Further confirmation of *Lactobacillus* and *Gardnerella*’s association with infertility in Bangladeshi populations offers valuable insights into the global significance of such biomarkers as predictors of infertility.

Unlike most other *Lactobacillus* species, *Lactobacillus iners* produces only l-lactic acid, which is less effective in preventing the progression of pathogenic bacteria during vaginal infection compared to d-lactic acid produced by other *Lactobacillus* species (Zheng et al., 2021). This difference leads to a higher L/D ratio in *L. iners*, triggering the activation of metalloproteinase-8, which aids in breaking down the extracellular matrix (Beghini et al., 2015). This breakdown helps bacteria cross the cervix, leading to infections in the upper genital tract. Unlike other *Lactobacillus* species, *L. iners* also doesn’t produce hydrogen peroxide (H_2_O_2_) (Chee et al., 2020), which is a key defense mechanism against anaerobic bacteria in the vagina. Due to these factors, *L. iners* is less effective in preventing the invasion of pathogens and the development of vaginal dysbiosis, potentially leading to infertility (Zheng et al., 2021).

Furthermore, *Gardnerella vaginalis* is known to trigger the production of proinflammatory cytokines like IL-12 (p70), IL-8, IL-1b, and IL-1a in the vagina, potentially affecting the viability of sperm (Chen et al., 2021). This bacterium carries the *Sialidase A* gene, which is linked to Bacterial Vaginosis and the formation of biofilm. By using sialidase, *G. vaginalis* breaks down sialic acid in vaginal mucus, weakening its protective barrier. Additionally, it produces vaginolysin, a toxin that creates pores in vaginal cells, making them more vulnerable to infection. These factors enable viruses and bacteria to invade and thrive in the vagina, contributing to infertility (Morrill et al., 2020; Chen et al., 2021). Therefore, the increased abundance of both *Lactobacillus iners* and *Gardnerella vaginalis* in the disease group suggests a potential association with infertility. Additionally, *Lactobacillus gasseri*, which is linked to DNA fragmentation in oocytes and reduced mobility of sperm, leading to infertility (Campisciano et al., 2017), was found to be more abundant in the disease group in our study, further suggesting its correlation with infertility.

In contrast, the control samples exhibit greater diversity in terms of species composition. Out of the 5 samples from the control group, one sample was dominated by *Atopobium vaginae*, another by *Lactobacillus helveticus*, one by *Janthinobacterium lividum* group (Proteobacteria), one by *Staphylococcus aureus* group, and another by *Lactobacillus iners*, representing CST-4B, CST-1, CST-4C, CST-4C, and CST-3, respectively. While *Staphylococcus aureus* and *Janthinobacterium lividum* group have been previously found in the healthy women’s vagina (Babu, 2017; Das Purkayastha et al., 2019), its noteworthy that *Atopobium vaginae* is linked to bacterial vaginosis (Polatti, 2012).

Overall, both groups were characterized by dominance at the species level, with *Lactobacillus iners*, *Lactobacillus helveticus*, *Lactobacillus gasseri*, and *Gardnerella vaginalis*. However, the diseased group exhibited higher abundances of these bacteria compared to the control group. Among these, the association of *Lactobacillus iners*, *Lactobacillus gasseri*, and *Gardnerella vaginalis* with infertility is well supported by previous research (Campisciano et al., 2017, 2020; Gholizadeh et al., 2023).

The alpha diversity of the samples from the two groups was analyzed to compare species richness, evenness, and diversity using various alpha diversity metrics such as Chao1, ACE, Shannon, Simpson, etc. All metrics consistently indicated that the disease group exhibited higher species richness but lower diversity. Conversely, the control group showed the opposite pattern. This observation may be attributed to the elevated abundance of specific species, such as *Lactobacillus iners*, *Lactobacillus helveticus*, and *Gardnerella vaginalis*, in the disease group. Observed higher species richness within the infertile group aligns with findings from earlier research (Campisciano et al., 2017). These dominated or depleted microbiota may affect fertility through several potential mechanisms. Firstly, the dominance of *Lactobacillus* species, especially *Lactobacillus iners* and *Lactobacillus helveticus*, in the disease group may lead to higher species richness but lower diversity. Conversely, the lower species richness but higher diversity observed in the control group may indicate a more balanced and stable vaginal microbiota composition, conducive to optimal reproductive health. The prevalence of *Gardnerella vaginalis* in the vaginal microbiota of infertile individuals may hinder fertility through inflammation, altered vaginal pH, and disruption of the vaginal epithelium. *Gardnerella vaginalis* also causes endometriosis, which is a major factor in causing infertility (Khan et al., 2014). Further investigation is needed to elucidate the specific mechanisms by which these microbiota alterations influence fertility.

The beta diversity analysis, which assesses diversity dissimilarity among samples, did not show the expected formation of two distinct clusters representing the disease and control groups. Instead, the analysis identified four distinct clusters, with three of them containing samples from both the disease and control groups, while one cluster was unique, exclusively composed of two healthy samples.

In-depth shotgun metagenomic sequencing of the samples not only unveiled the non-bacterial burden but also cross-verified the bacterial taxonomy. The possibility of sub-clinical infections in apparently healthy samples, potentially sharing a similar microbiome with diseased samples, could explain the observed lack of significant differences in bacterial composition between the two sample types, as indicated by diversity metrics. To address this, we included 10 publicly available healthy vaginal metagenomic samples in the study, enhancing the robustness of our comparative analysis.

While both the disease and control groups are dominated by *Lactobacillus iners*, mirroring the findings from 16S amplicon sequencing, their abundances differ. *Lactobacillus iners* constitutes 71.6% of the microbiota in the disease group, whereas it comprises 54.9% in the control group. In contrast, public datasets exhibit dominance by a more diverse group of microorganisms, where *Lactobacillus* is not the prevailing flora. Thus, the presence of *Lactobacillus iners* might indicate vaginal dysbiosis and the possibility of infertility.

Fungi are exclusively found in the control group. *Penicillium citrinum* is the dominant among the fungal species identified. No significant difference was observed in the abundance of viruses between the disease and control groups, both comprising approximately the same amount of Human endogenous retrovirus K113 and other viruses. Interestingly, public datasets exhibit less than half a percentage of this virus compared to these two groups. Therefore, exploring their potential relationships with infertility is not beyond consideration.

Taxonomy-independent analysis involved clustering of contigs constituting metagenomically-assembled genomes (MAGs). Species identification within the MAGs revealed greater diversity in the control group, featuring two additional clusters containing *Penicillium* and *Staphylococcus haemolyticus* compared to the disease group (**Figure 9A**). Interestingly, these clusters are also present in public datasets (**Figure 9B**). In contrast, the disease group exhibited only three clusters, including one with Human endogenous retrovirus K113 and Shamonda orthobunyavirus, another with *Lactobacillus*, and a third with *Gardnerella*. While the control group also harbors these clusters, the disease group displayed a higher number of contigs (**Figure 9C**). The increased richness of these specific microorganisms in the disease group suggests a potential link with infertility.

Infertility is a complex phenomenon influenced by numerous factors, among which vaginal dysbiosis might play a role. This study suggests a potential relationship between the overabundance of certain microorganisms, such as *Lactobacillus iners, Lactobacillus gasseri* and *Gardnerella vaginalis*, and female infertility. However, it is important to acknowledge several limitations of this study. The small sample size hinders reaching a conclusive decision, and the lack of well-understood clinical pathophysiology further complicates interpretation. Additionally, factors such as ethnicity, race, and geographical diversity in the patient population can significantly affect the overall microbiome diversity, potentially impacting study outcomes. Despite these limitations, this study represents an initial effort to explore the relationship between vaginal microflora and infertility in a resource-limited setting. Moving forward, larger-scale studies with diverse patient populations are necessary to provide more robust data and insights into this complex association.

## Data availability statement

The datasets presented in this study can be found in online repositories. The names of the repository/repositories and accession number(s) can be found below: https://www.ncbi.nlm.nih.gov/, SAMN39995661, SAMN39995662, SAMN39995663, SAMN39995664, SAMN39995665, SAMN39995666, SAMN39995667, SAMN39995668, SAMN39995669, SAMN39995670.

## Ethics statement

The studies involving humans were approved by Faculty of Biological Sciences, University of Dhaka. The studies were conducted in accordance with the local legislation and institutional requirements. The participants provided their written informed consent to participate in this study. Written informed consent was obtained from the individual(s) for the publication of any potentially identifiable images or data included in this article.

## Author contributions

ZH: Data curation, Formal analysis, Investigation, Methodology, Writing – original draft, Writing – review & editing. MN: Data curation, Formal analysis, Investigation, Software, Writing – review & editing. NB: Investigation, Methodology, Writing – review & editing. NH: Supervision, Validation, Visualization, Writing – review & editing, Formal analysis, Software. MY: Conceptualization, Funding acquisition, Supervision, Validation, Writing – review & editing. SA: Conceptualization, Funding acquisition, Project administration, Supervision, Validation, Writing – review & editing.
